# Dissociable contributions of mediodorsal and anterior thalamic nuclei in visual attentional performance: A comparison using nicotinic and muscarinic cholinergic receptor antagonists

**DOI:** 10.1177/0269881120965880

**Published:** 2020-10-24

**Authors:** Craig P Mantanona, Tadej Božič, Yogita Chudasama, Trevor W Robbins, Jeffrey W Dalley, Johan Alsiö, Ilse S Pienaar

**Affiliations:** 1 Faculty of Health and Life Sciences, Northumbria University, Newcastle upon Tyne, UK; 2 Department of Psychology, University of Cambridge, Cambridge, UK; 3 Section on Behavioral Neuroscience, National Institute of Mental Health, Bethesda, USA; 4 Department of Psychiatry, Hershel Smith Building for Brain and Mind Sciences, Forvie Site, Cambridge, UK; 5 School of Life Sciences, University of Sussex, Brighton, UK

**Keywords:** Acetylcholine, attention, muscarinic receptors, nicotinic receptors, prefrontal cortex, thalamus

## Abstract

**Background:**

Thalamic subregions mediate various cognitive functions, including attention, inhibitory response control and decision making. Such neuronal activity is modulated by cholinergic thalamic afferents and deterioration of such modulatory signaling has been theorised to contribute to cognitive decline in neurodegenerative disorders. However, the thalamic subnuclei and cholinergic receptors involved in cognitive functioning remain largely unknown.

**Aims:**

We investigated whether muscarinic or nicotinic receptors in the mediodorsal thalamus and anterior thalamus contribute to rats’ performance in the five-choice serial reaction time task, which measures sustained visual attention and impulsive action.

**Methods:**

Male Long-Evans rats were trained in the five-choice serial reaction time task then surgically implanted with guide cannulae targeting either the mediodorsal thalamus or anterior thalamus. Reversible inactivation of either the mediodorsal thalamus or anterior thalamus were achieved with infusions of the γ-aminobutyric acid-ergic agonists muscimol and baclofen prior to behavioural assessment. To investigate cholinergic mechanisms, we also assessed the behavioural effects of locally administered nicotinic (mecamylamine) and muscarinic (scopolamine) receptor antagonists.

**Results:**

Reversible inactivation of the mediodorsal thalamus severely impaired discriminative accuracy and response speed and increased omissions. Inactivation of the anterior thalamus produced less profound effects, with impaired accuracy at the highest dose. In contrast, blocking cholinergic transmission in these regions did not significantly affect five-choice serial reaction time task performance.

**Conclusions/interpretations:**

These findings show the mediodorsal thalamus plays a key role in visuospatial attentional performance that is independent of local cholinergic neurotransmission.

## Introduction

The thalamus is segregated into many distinct nuclei based on neural connectivity, as well as functional and neurochemical attributes. These subnuclei contribute to large-scale networks associated with specific aspects of cognitive function. In addition to causing mnemonic deficits (Dalrymple-Alford et al., 2015; Leszczyński and Staudigl, 2016), lesions affecting the thalamus also result in a range of executive deficits associated with attention, working memory, cognitive flexibility and decision making (Linley et al., 2016; Parnaudeau et al., 2018; Wright et al., 2015), with these functions likely to depend on distinct subregions of the thalamus. In rodents, the mediodorsal (MD) and anterior thalamic (AT) nuclei have received significant attention due to their distinct cortical inputs. For example, the AT is critical for regulating spatial aspects of memory performance by virtue of its strong hippocampal connections (O’Mara and Aggleton, 2019). In contrast, the MD is thought to be more relevant for executive type functions due to its interactions with the prefrontal cortex (PFC) (Bueno-Junior et al., 2018; Parnaudeau et al., 2015). Consequently, when tested on frontal-type tests associated with sustained and spatially divided visual attention, rats with MD lesions increase impulsive, premature responding, whereas those with AT lesions show no change in most aspects of attentional performance (Chudasama and Muir, 2001).

The MD and AT also receive a dense cholinergic innervation from the nucleus basalis magnocellularis (NBM) and from various brainstem nuclei including the peduncolopontine tegmental (PPTg) and laterodorsal tegmental (LDTg) nuclei (Pienaar et al., 2017; Soares et al., 2018). In this regard, cholinergic lesions and anticholinergic drugs compromise performance in tasks that assess visual attention and signal detection (Cyr et al., 2015; Harati et al., 2008; Ljubojevic et al., 2018; Risbrough et al., 2002). Given the NBM sends direct cholinergic input to the PFC, the resulting attentional deficits may reflect reduced cholinergic tone in this brain region. However, what remains unknown is whether acetylcholine (ACh) receptor signaling in the MD and AT contribute to attentional function as well. If cholinergic innervation of the MD and AT differentially affects sustained and selective forms of visual attention, it would suggest these two thalamic nuclei provide a mechanism by which they can potentially modulate prefrontal control of attention.

In the current study we used the five-choice serial reaction time task (5-CSRTT) to assess attentional function in rats following (a) temporary inactivation of the MD and AT through a local infusion of a drug cocktail consisting of muscimol, an agonist for the γ-aminobutyric acid (GABA)_A_ receptor, and baclofen, which acts as an agonist to the GABA_A_ receptor, and (b) by locally microinfusing scopolamine (a muscarinic cholinergic receptor antagonist) and mecamylamine (a nicotinic cholinergic receptor antagonist) to block cholinergic transmission in the MD and AT in a receptor-subtype-specific manner.

## Methods

### Animals

A total of 24 male Long-Evans rats (Charles River Ltd., Margate, UK) were initially used in this study. The animals were housed in groups of 3–4 per cage, with a reversed light/dark cycle applied (lights off between 7:00 and 19:00 h). Behavioural testing occurred during the dark phase of this cycle. Animals were food restricted at 18 g of laboratory pellet chow once a day post training/testing, but with ad libitum access to water. The rats’ weights were monitored to remain above 85% of their free-feeding body weight. All experimental procedures complied with the UK Animal (Scientific Procedures) Act 1986 and were approved by the Local Ethics Review Committee (University of Cambridge, UK). Both the Guide for the Care and Use of Laboratory Animals (1996) (National Research Council, 2011) and the Animals in Research: Reporting In Vivo Experiments guidelines (Kilkenny et al., 2010) were followed for all animal research procedures performed for the current work.

### 5-CSRTT

The 5-CSRTT is a well-validated behavioural paradigm for assessing sustained spatial visual attention in rats (Mantanona et al., 2019). The rats were trained and tested in Bussey-Saksida Touch Screen chambers (Lafayette Instrument, Indiana, USA) controlled by ABET II and Whisker control software (Cardinal and Aitken, 2010). Chambers were trapezoidal (30 cm height × 35 cm length × 30 cm front end width × 25 cm rear end width) with a touch-sensitive liquid-crystal display flat screen placed at the front end. The food magazine was located at the rear of the chamber and dispensed 45 mg reward sugar pellets (Sandown Scientific, Middlesex, UK) from an external dispenser. Black Perspex masks with five evenly spaced square apertures located basally in the rat’s immediate field of vision were secured in front of the touchscreens.

**Figure 1.**
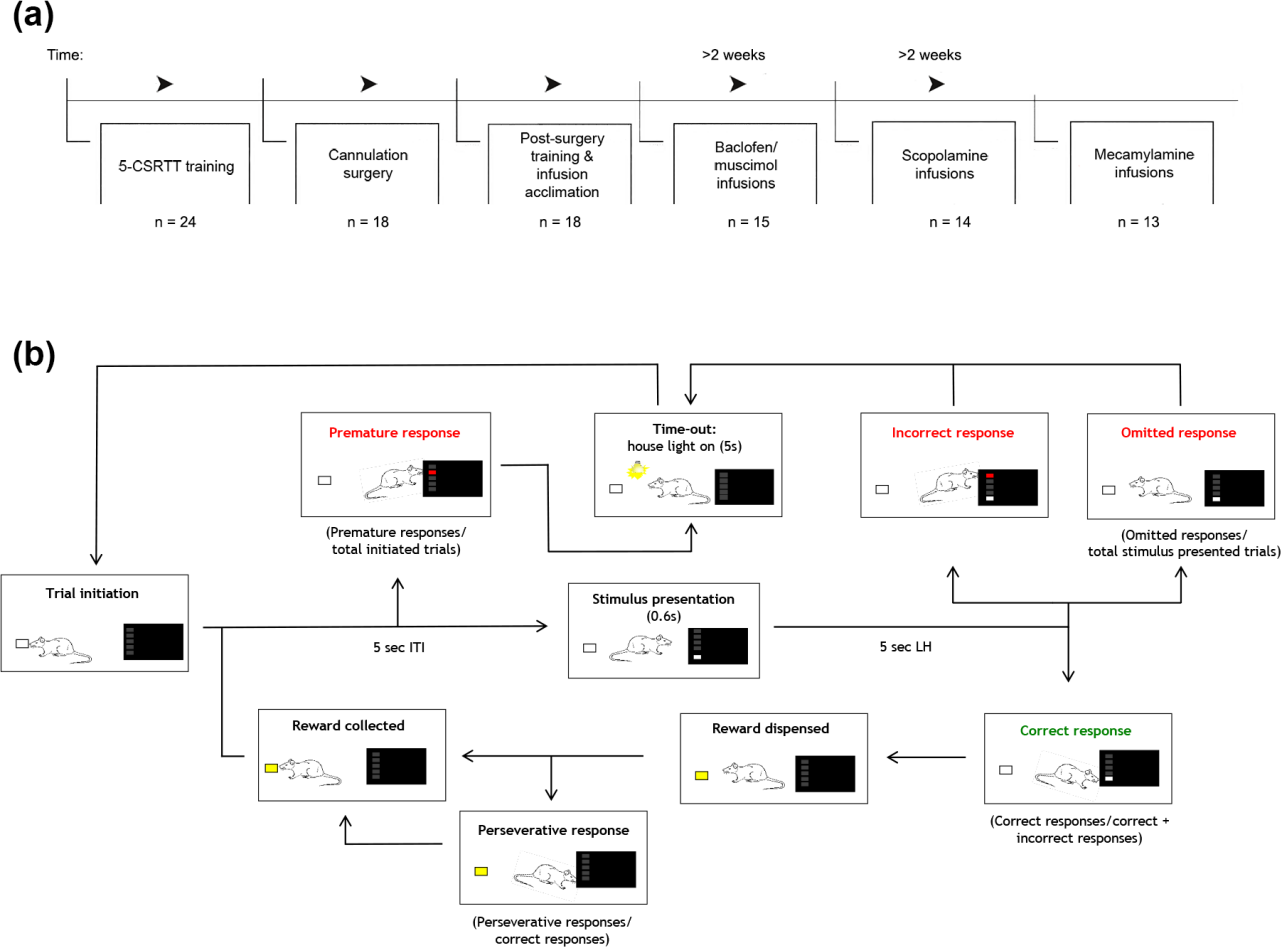
(a) Experimental sequence for training in the five-choice serial reaction time task (5-CSRTT) and related drug-testing experiments. (b) A diagram representation of the 5-CSRTT test protocol. For 5-CSRTT testing, a trial was initiated when a rat nose-poked into the food magazine located at the rear of the chamber. By making a ‘premature’ nose-poke prior to stimulus presentation, a time-out punishment was initiated, consisting of the light being on for 5 s, preventing the rat from performing an action, followed by the trial commencing again. Stimulus is presented after a 5 s inter-trial interval (ITI). An ‘incorrect’ nose-poke response initiated a time-out punishment. A correct response was rewarded with a sugar pellet in the food magazine. Continuous responses into the same correct response aperture prior to reward collection was counted as perseverative responses. The next trial was initiated following reward collection.

A standard touchscreen-based 5-CSRTT protocol was followed, as shown in Figure 1(a) and (b), and described previously (Bharmal et al., 2015; Mantanona et al., 2019; Nilsson et al., 2016). Animals were trained to nose poke into a briefly illuminated, spatially random aperture. The training and baseline task ended either after 30 mins or when animals completed 100 trials. At the end of the training, the stimulus duration (SD) was set to 0.6 s and the inter-trial interval (ITI) to 5 s. The variables recorded at each session included: (a) number of correct responses; (b) number of incorrect responses; (c) accuracy, that is, the number of correct responses divided by the total number of correct and incorrect responses; (d) number of omitted responses for all trials where a stimulus was presented; (e) number of premature responses made during the ITI for all trials initiated; (f) number of perseverative responses – repeat responses made after a correct response; (g) correct response latency – time from stimulus presentation until a correct response was made; (h) incorrect response latency – time from stimulus presentation until an response was made on incorrect trials; and (i) magazine latency – time to collect reward post correct response.

### Surgical procedures

Animals were anaesthetised with 3–5% isoflurane vaporized in 2.0 l/min O_2_. Analgesia was provided by subcutaneous administration of 0.2 mg/kg Meloxicam (Metacam^®^, Boehringer Ingelheim, Bracknell, UK). Individual rats were then secured in a stereotaxic frame (Kopf, Tujunga, CA, USA). To prevent hypothermia, rats were placed on a homeostatic heating pad with body temperature monitoring via rectal sensor. For the surgery, a small, elliptical patch of scalp was removed along the midline of the skull to accommodate the cannulae, which were inserted bilaterally. Four stainless steel screws were placed into the skull, two lying anterior and two lying posterior to the guide cannula insertion sites. After attaining a flat skull position, a dental drill was used to drill two bilateral holes through the cranium. Through each hole a stainless-steel guide cannula (22 gauge; PlasticsOne, Cleveland, OH, USA) was inserted at a 24° angle to target the MD, using the following stereotaxic coordinates: −2.52 mm anteroposterior (AP) to bregma, ±3.4 mm mediolateral (ML) and −2.7 mm dorsoventral (DV) from the dura. For targeting the AT we used the coordinates −1.80 mm AP to bregma, ±3.4 mm ML and −2.7 mm DV from the dura. To hold the cannulae in place, dental acrylic cement was applied around the cannulae and screws. Each guide cannula had a dummy cannula inserted within the internal lumen and a protective cap, which remained in situ until the drug infusions. Post-surgical analgesia (1 mg/kg, Metacam^®^) was orally administered for 3 consecutive days post-surgery. Animals were given at least 7 days to recover from the surgery before behavioural training resumed.

### Drug preparation and infusions

Once the rats had consistently achieved baseline criteria on the behavioural task (⩾70% accuracy and ⩽20% omissions), vehicle (saline) infusions were given to habituate rats to infusions. Testing began once all rats reached baseline criteria with vehicle infusion. Rats were then tested across three separate and consecutive Latin squares, using muscimol/baclofen, scopolamine and finally mecamylamine. Then 1 day of re-baseline testing (without any drug infusions) was included before each drug treatment regime was initiated. In addition, animals were left for a minimum of 14 days between each Latin square; baseline sessions were included during this period to ensure stable performance.

The GABA_A_ receptor agonist, muscimol hydrobromide (Sigma-Aldrich, Dorset, UK) and baclofen hydrochloride, a GABA_B_ receptor agonist (Sigma-Aldrich), as well as the muscarinic antagonist scopolamine hydrobromide (Sigma-Aldrich) and nicotinic antagonist, mecamylamine hydrochloride (Sigma-Aldrich) were administered to the rats. All drug solutions were prepared by dissolving the drug in physiological saline. Muscimol and baclofen were prepared as a cocktail of equimolar amounts of both compounds: either 0.01 nmol or 0.03 nmol per infusion volume per cerebral hemisphere (a total volume of 0.3 µl was given). Scopolamine was initially prepared in concentrations of 3, 6, and 10 µg/0.3 µl; however, at the highest dose (10 µg scopolamine) performance on the task was severely disrupted in both MD- and AT-infused rats, hence the final Latin square did not include this dose. Mecamylamine was prepared in concentrations of 3, 6, and 10 µg/0.3 µl. Previously published work reporting on performance-altering effects seen in rodent behavioural tasks (Mitchell et al., 2002; Riekkinen et al., 1995) made use of a similar dose (10 µg/0.3 µl), which comprised the highest dose used in the current study, for both the muscarinic and nicotinic antagonists, scopolamine or mecamylamine.

Drug infusions were made using a syringe pump (Harvard Apparatus UK, Cambourne, UK) with two 10 µl Hamilton syringes connected via polythene tubing (0.28 mm inner diameter, 0.61 outer diameter; Smiths Medical, Ashford, UK) to the internal cannulae, which extended 3 mm below the guides. After the internal cannulae were inserted into the brain, we allowed 60 s before infusions began; drug or vehicle was then delivered at a rate of 0.3 µl/min over 1 minute. Another 60 s was allowed post-infusion for the diffusion of the drugs, before the internal cannulae were slowly removed and the dummy cannulae were returned. Each rat included in the study received a maximum of 24 infusions (to deliver either the vehicle or drug) for the whole duration of the study.

### Histology assessment of cannulae placements

Following completion of the behavioural experiments, rats were euthanised with a lethal dose of sodium pentobarbital (Euthatal^®^, 200 mg/ml, Merial, UK) before being transcardially perfused with 0.01 M phosphate buffered saline (PBS), followed by 4% paraformaldehyde (PFA). Brains were harvested and post-fixed overnight in 4% PFA, followed by 30% sucrose in 0.01 PBS until brains were sufficiently dehydrated. Brains were sectioned coronally at 30 µm thickness and then stained with cresyl fast violet (CFV) for labelling neurons containing Nissl substance. In brief, the staining procedure involved incubating the slides in 0.5% cresyl violet acetate (Sigma-Aldrich) for 3 min, differentiated in 70% absolute ethanol, dehydrated through an ascending graded ethanol series, cleared in xylene, and finally cover slipped. From the histology slides we confirmed the cannula placements by consulting a rat brain atlas (Paxinos and Watson, 2004), with such assessments performed by an investigator blind to the treatment of the rats.

### Statistical analysis

Data analysis for number of trials, accuracy, errors of omission, premature responses, number of perseverative responses and latencies were conducted using SPSS statistical software (v. 24, SPSS Inc., Chicago, IL, USA). Normality of residuals were assessed using Q-Q plots and Shapiro-Wilks test. Non-normally distributed data were transformed appropriately using arcsine, square root and logarithmic transformations (Winer, 1971). Mauchly’s sphericity test was used to assess the homogeneity of variance across datasets. If datasets violated this requirement, data were transformed, as detailed in the Results section, with a conservative Greenhouse-Geisser correction used to correct degrees of freedom. A mixed-effects analysis of variance was used to analyse the within-subject effects of drug dose, the between-subject effects of brain region and the dose × brain region interactions. Post-hoc analyses were carried out using the Bonferroni correction for multiple comparisons. Power estimates are shown in Supplementary Table 1. Throughout, *p<*0.05 was considered significant. Data are expressed as mean ± standard error of the mean (SEM). Five rats were excluded from surgery and further testing due to their inability to maintain a stable level of performance. A further three rats were excluded due to an inability to reach criteria performance after surgery. In addition, one rat died of unknown causes in the home cage before surgery.

## Results

**Figure 2.**
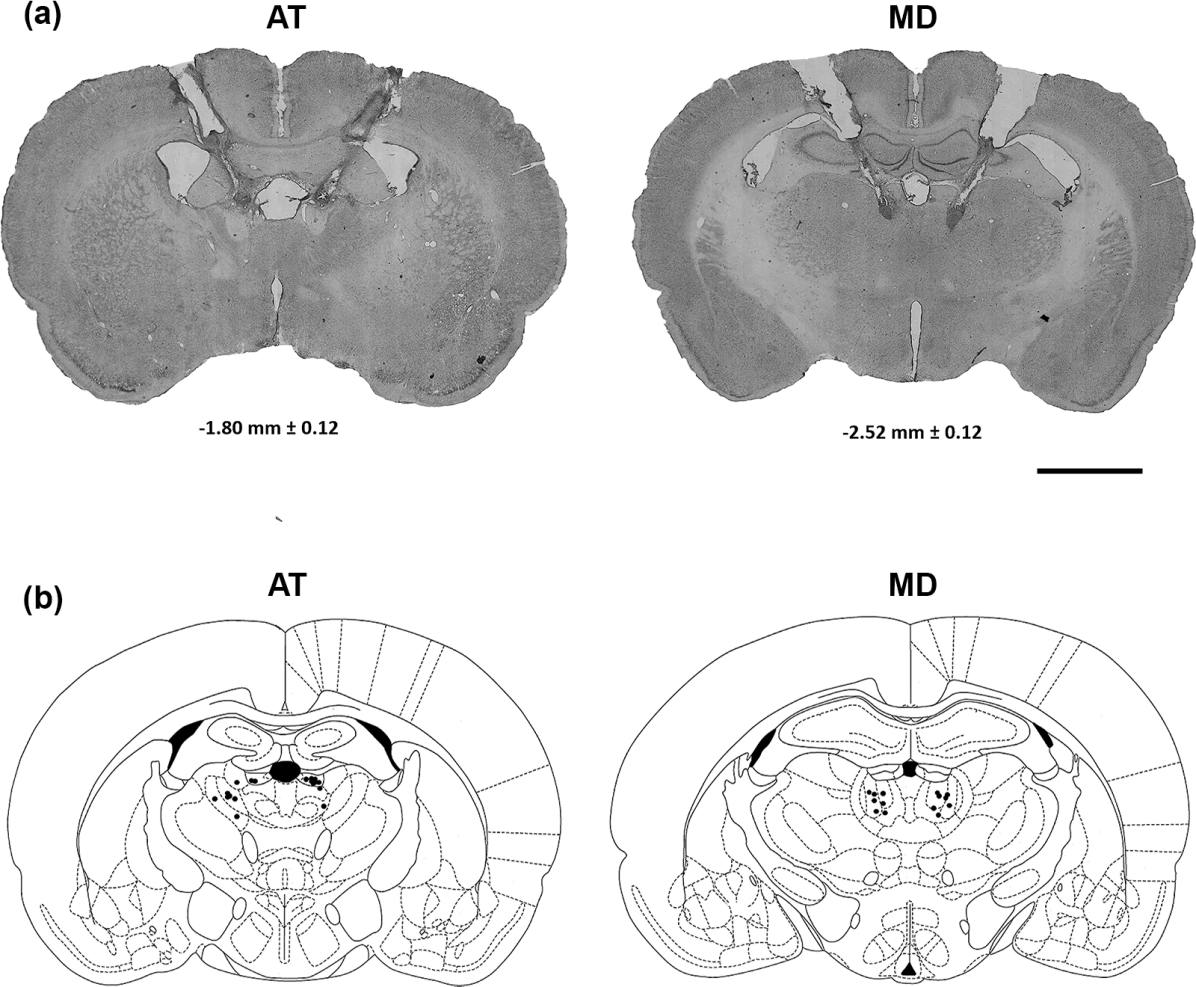
(a) Representative cresyl fast violet (CFV)-stained sections reveal the cannulation and injection needle tracks for targeting the anterior thalamus (AT) and mediodorsal (MD), (b) corresponding to the respective neuro-anatomical schematics (taken from Paxinos and Watson, 2004) for the AT and MD infusion sites. Confirmed bilateral infusion sites are indicated as black dots. In general, inspection of all histologically stained brain sections, representing the cohort of rats used in the current study, showed the brain tissue had recovered well from the repeated drug injection protocols, with no evidence seen of major scar tissue formation (reactive gliosis). Scale bar: 400 μm.

Histological assessment revealed that cannula tips were distributed uniformly in the medial, central and lateral regions of the MD. Cannula tips in the group of rats in which the AT had been inactivated were largely restricted to the anterodorsal thalamus, the dorsomedial aspect of the anteroventral thalamus and the stria medullaris. Representative CFV-stained sections are depicted in Figure 2(a) and (b) to illustrate placement of cannulation and injection needle tracks for targeting the AT and MD in the rats.

### Inactivation of the MD produces marked deficits in visuospatial attention

**Figure 3.**
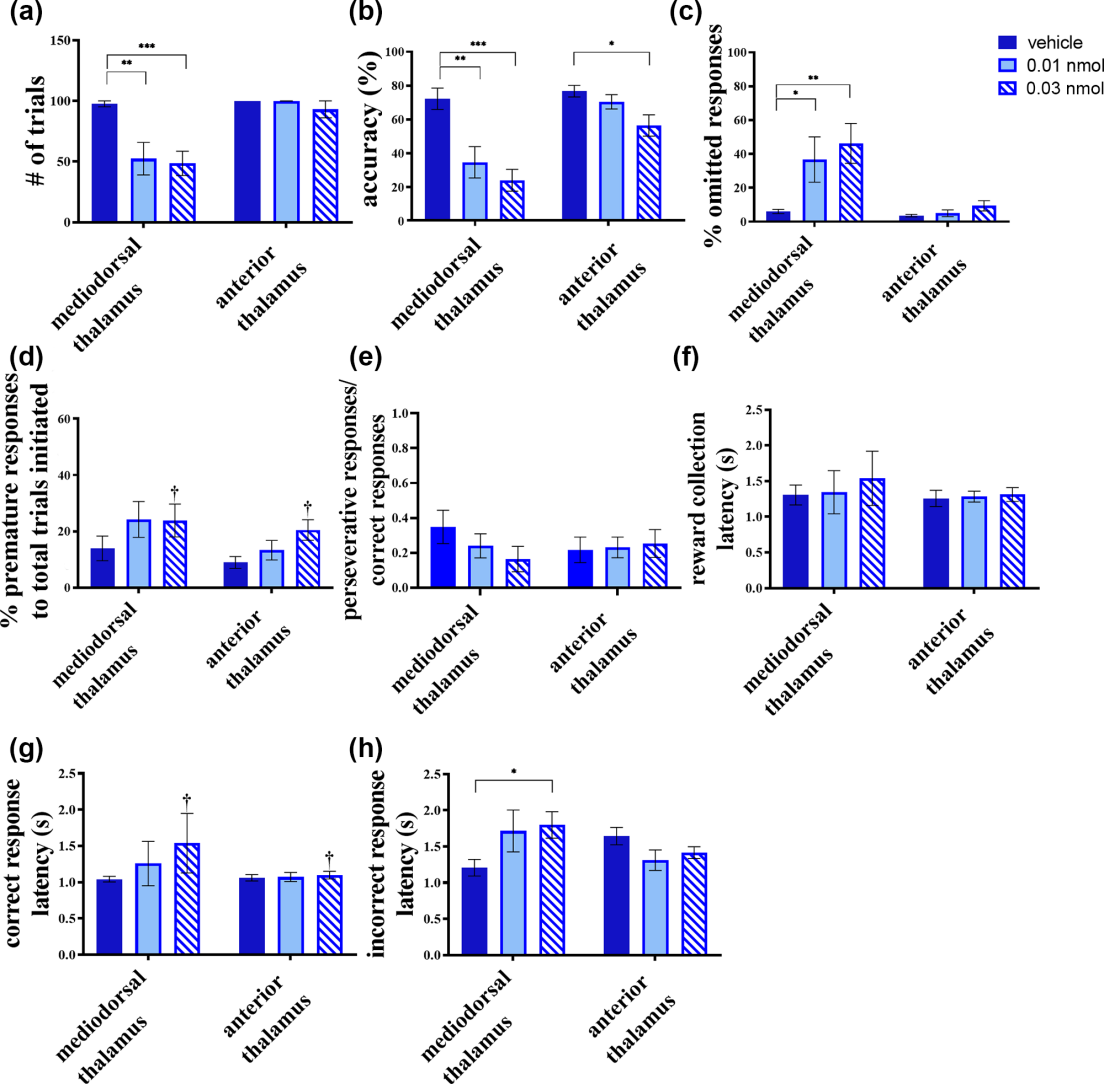
Effects of baclofen/muscimol microinfusions into the mediodorsal (MD) and the anterior thalamus (AT) during testing in the five-choice serial reaction time task (5-CSRTT). Attentional deficits resulted from MD infusions, which manifested as a decrease in (a) trials and (b) accuracy and an increase in the (c) percentage responses omitted. (d) Rats in both groups showed a trend to respond prior to stimulus presentation following baclofen/muscimol infusions. Such premature responses are interpreted as an index for impulsivity. Although a main effect of treatment was seen in premature responses, no significant interaction of dose × region was found. (e) The ratio of perseverative responses:correct responses showed no significant difference when comparing treatment groups, indicating that any significant decrease in perseverative responses was likely caused by a decrease in correct responses. (f) Reward collection latency remained intact in both regions following all microinfusions. (g) A main effect of dose was seen on correct response latency, but no significant interaction was found. (h) Incorrect response latency was increased significantly by the high dose in MD infusions with no other significant effects seen for either thalamic regions. Values are shown as mean ± SEM. Significance is denoted as *p <0.05, **p<0.01, ***p<0.001. Significant main effect of dose-only is denoted as ^†^*p* <0.05.

Inactivation of the MD (*n*=7) had a large impact on rats’ performance in the 5-CSRTT relative to inactivation of the AT (*n*=8) (Figure 3). One major change between the two groups was the number of trials completed (dose × region interaction; *F*(2, 26)=9.449; *p=*0.001; square root transformation). Those rats with MD inactivations completed fewer trials at both the low (0.01 nmol; *p=*0.001) and high (0.03 nmol; *p<*0.001) doses (Figure 3(a)). In contrast, inactivating the AT had no such effect on trials completed (*p*>0.1). Consequently, all further analyses were adjusted for total trials initiated or completed, as appropriate.

The effect of muscimol/baclofen treatment on accuracy depended on the thalamic subregion being targeted (Figure 3(b)), indicated by the dose × region interaction (*F*(2, 26)=5.852, *p=*0.008). Post-hoc analysis confirmed that MD inactivations reduced accuracy at both low (*p=*0.001) and high (*p*<0.001) doses of muscimol/baclofen, whereas AT inactivations only impaired accuracy at the high dose (*p=*0.041). Accompanying the reduced accuracy was an increase in the percentage omissions (dose × region interaction: *F*(1.411, 18.356)=4.565, *p=*0.035; arcsine root transformation) (Figure 3(c)) exhibited by the MD-inactivated group; such animals showed a percentage of omissions following both low (*p=*0.030) and high (*p=*0.002) doses of muscimol/baclofen. This effect was not detected following intra-AT infusions (*p*>0.1).

Muscimol/baclofen treatment produced a main effect of dose, revealed as increase in the percentage premature responses (*F*(2, 26)=4.418, *p=*0.022; arcsine root transformation) shown by both the MD and AT groups, but no significant dose × region interaction (*F*(2, 26)=0.343, *p*>0.1). Post-hoc analysis revealed the high dose of muscimol/baclofen increased the percentage of premature responses (*p=*0.048) (Figure 3(d)). The ratio of perseverative responses was unaffected by the dose (*F*(2, 26)=1.223, *p>*0.1; Figure 3(e)), whereas no dose × region interaction was detected (*F*(2, 26)=2.692, *p>*0.1).

Muscimol/baclofen-mediated thalamic inactivation did not alter the latency to collect reward, in accordance to the given dose (*F*(2, 26)=2.038, *p*>0.1; logarithmic transformation) (Figure 3(f)) for either the MD- or AT-inactivated group, with no dose × region interaction seen (*F*(2, 26)=0.723, *p*>0.1). Correct response latencies increased as a main effect of dose (*F*(2, 26)=3.981, *p=*0.031; logarithmic transformation) (Figure 3(g)); however, no significant dose × region interaction was seen, especially at the high dose (*p*=0.059). In addition, incorrect response latency analysis revealed a dose × region interaction (*F*(2, 26)=4.321, *p=*0.024; logarithmic transformation) (Figure 3(h)). Post-hoc analysis confirmed the increase in this incorrect latency occurred in the MD-inactivated group at the high dose only (*p=*0.037).

### Cholinergic receptor antagonism of the MD and AT does not affect attention

**Figure 4.**
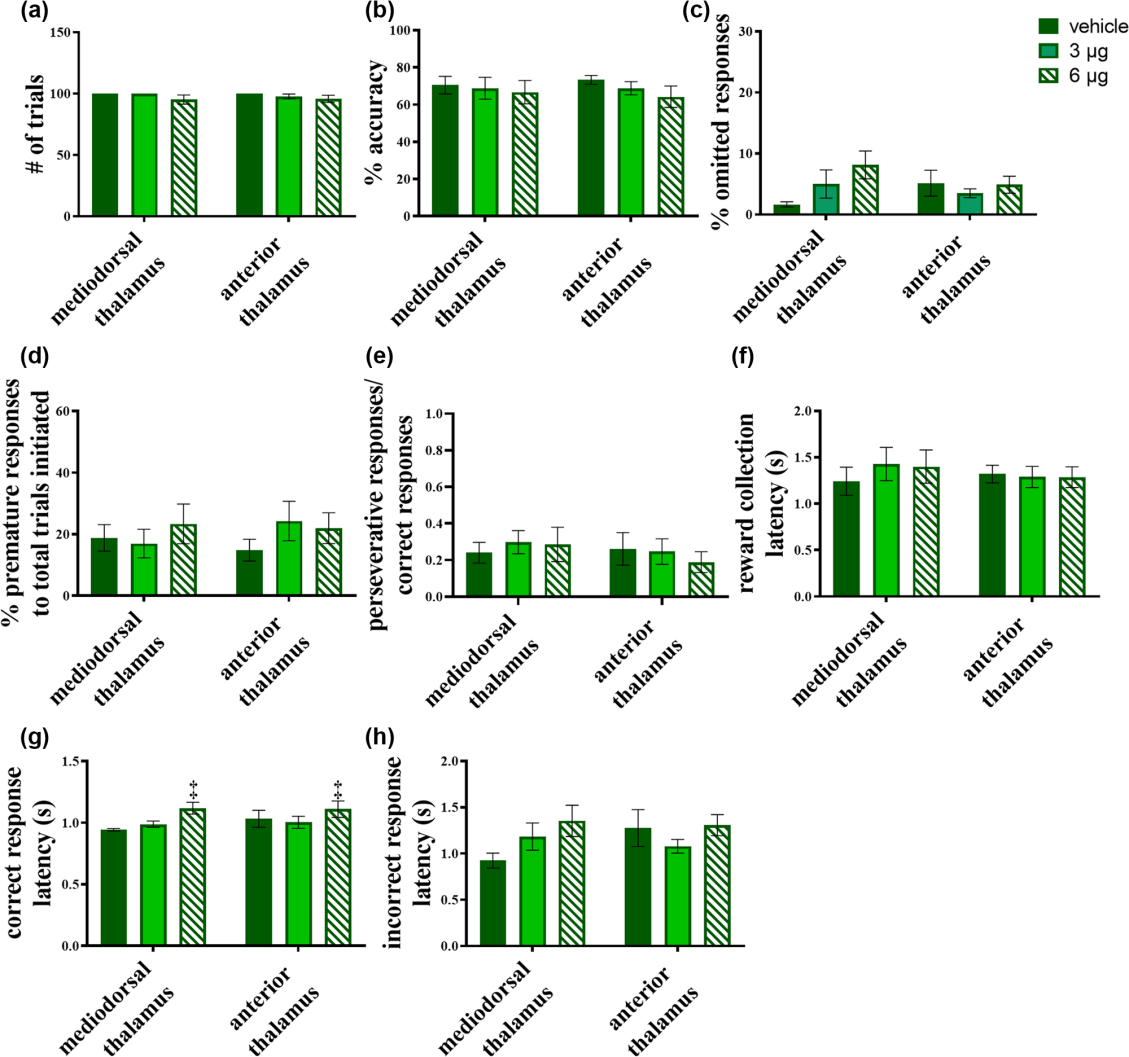
Effects of scopolamine microinfusions into the mediodorsal (MD) and the anterior thalamus (AT) on five-choice serial reaction time task (5-CSRTT) performance. No significant effect was produced in (a) the number of trials or (b) accuracy of responses. (c) The percentage of omitted responses was similarly unaffected in either region by all doses. These results support a lack of effect on sustained attention by muscarinic agonism in the MD and AT. (d) The percentage premature responses were also left unaffected, with similar results for (e) the ratio of perseverative responses:correct responses, indicating that scopolamine did not alter aspects of inhibitory control. (f) There was no effect on reward collection latency. (g) Scopolamine infusions produced a main effect of dose on correct response latency, but no significant interaction of dose × region was seen. (h) Incorrect response latencies were unaffected by infusions into either thalamic region. Significant main effect of dose-only is denoted as *p*<0.005.

One rat from the MD group was removed from all further cholinergic manipulations due to consistently poor baseline performance. Microinfusions of the muscarinic ACh receptor antagonist, scopolamine into the MD (*n*=6) and AT (*n*=8) caused no significant change in the animals’ performance. Figure 4(a) shows that scopolamine did not affect the number of trials completed (*F*(1.365, 16.385)=2.723, *p*>0.1; square root transformation); dose × region interaction: *F*(1.365, 16.385)=0.267, *p*>0.1). Other parameters were also not affected for either group, including performance accuracy (main effect of dose: *F*(2, 24)=1.895, *p*>0.1; dose × region interaction: *F*(2, 24)=0.317, *p*>0.1) (Figure 4(b)) and percentage omitted responses (main effect of dose: *F*(2, 24)=2.098, *p*>0.1; dose × region interaction: *F*(2, 24)=2.446, *p*>0.1) (Figure 4(c)). Parameters reflecting inhibitory control were also unaffected for both MD and AT microinfused rats. These included percentage premature responses (main effect of dose: *F*(2, 24)=1.097, *p*>0.1; dose × region interaction: *F*(2, 24)=1.100, *p*>0.1) (Figure 4(d)) and the correct response ratio (main effect of dose: *F*(2, 24)=0.844, *p>*0.1); dose × region interaction: *F*(2, 24)=1.708, *p>*0.1) (Figure 4(e)).

In addition, scopolamine did not affect magazine latencies (main effect of dose: *F*(2, 24)=0.836, *p*>0.1; dose × region interaction *F*(2, 24)=2.276, *p*>0.1; logarithmic transformation) (Figure 4(f)), although it did slow correct responses in both the MD and AT groups (*F*(2, 24)=7.755, *p=*0.003; logarithmic transformation) (Figure 4(g)) at the highest dose (*p=*0.026). However, there was no scopolamine on incorrect response latencies; *F*(2, 24)=1.885, *p*>0.1; dose × region interaction: *F*(2, 24)=1.855, *p*>0.1) (Figure 4(h)).

**Figure 5.**
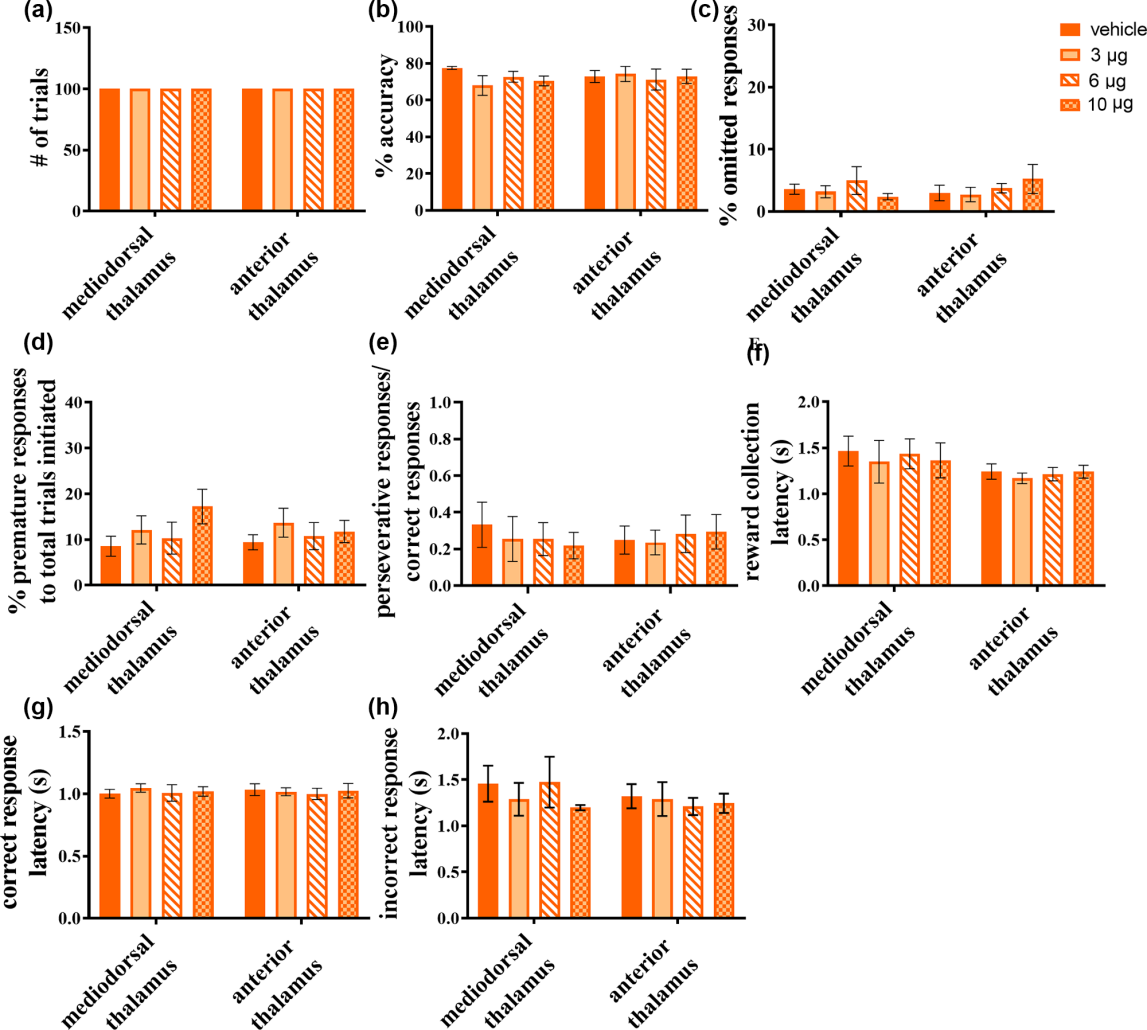
Effects of mecamylamine infusions into the mediodorsal (MD) and the anterior thalamus (AT) on rats’ five-choice serial reaction time task (5-CSRTT) performance. No effect was seen in (a) the number of trials completed, (b) response accuracy, or (c) the percentage of omitted responses. However, the drug was seen to have a significant main effect of dose on (d) percentage of premature responses, but this was not accompanied by a significant dose × region interaction. (e) The ratio of perseverative:correct responses showed no significant change from infusions. Similarly, (f) reward collection latency, (g) correct response latency and (h) incorrect response latency remained unaffected, with no significant effect of infusions. Significant main effect of dose-only is denoted as ^†^*p* <0.05.

The nicotinic receptor antagonist, mecamylamine, was also largely devoid of effects on the 5-CSRTT, regardless of the site of infusion (Figure 5). As shown in Figure 5(a), all rats, irrespective of infusion site, completed the maximum of 100 trials following mecamylamine infusions. One rat from the MD group was excluded because it failed to complete the counterbalanced sequence of mecamylamine and vehicle infusions leaving a sample size of *n*=5.

Mecamylamine infusions into the MD (*n*=5) and AT (*n*=8) did not affect accuracy (main effect of dose: *F*_3,33_=0.780, *p*>0.1; dose × region interaction: *F*_3,33_=1.309, *p*>0.1) (Figure 5(b)) nor percentage omitted responses (main effect of dose: *F*_3,33_=0.413, *p*>0.1; dose × region interaction: *F*_3,33_=0.840, *p*>0.1) (Figure 5(c)). However, there was a main effect of dose on the percentage of premature responses (*F*_3,33_=3.361, *p*=0.030), in the absence of a significant dose × region interaction (*F*_3,33_=1.158, *p*>0.1), whereas no significant effects were observed in post-hoc analysis when combining MD and AT grouped animals receiving the highest mecamylamine dose (*p*=0.153) (Figure 5(d)). Yet, visual inspection of the data suggests that mecamylamine administration increased the number of premature responses, with the MD group appearing more affected than the AT rats. In addition, mecamylamine did not affect perseverative responses at any level (main effect of dose: *F*(3, 33)=0.417, *p>*0.1; dose × region interaction *F*(3, 33)=1.367, *p>*0.1; Figure 5(e)).

Mecamylamine infusions did not affect several others aspects of performance including reward latency (main effect of dose: *F*(3, 33)=1.996, *p*>0.1; dose × region interaction: *F*(3, 33)=0.694, *p*>0.1) (Figure 5(f)), correct response latency (main effect of dose: *F*(3, 33)=0.502, *p*>0.1; dose × region interaction: *F*(3, 33)=0.399, *p*>0.1) (Figure 5(g)), nor incorrect response latency (main effect of dose: *F*(3, 33)=0.535, *p*>0.1; dose × region interaction: *F*(3, 33)=0.298, *p*>0.1) (Figure 5(h)).

## Discussion

In this study, we investigated performance in a touchscreen version of the 5-CSRTT following temporary inactivation of two associative thalamic nuclei, the MD and AT, and compared it with cholinergic antagonism of the muscarinic and nicotinic receptors using scopolamine or mecamylamine, respectively. Inactivating the MD severely disrupted discriminative accuracy as well as the speed and vigour of attentional performance. This was in marked contrast to inactivation of the AT, which produced only minor effects on performance that paralleled the lack of effects of nicotinic and muscarinic receptor antagonism in the MD and AT. The profile of deficits observed after MD inactivation indicates a critical role of this region in multiple aspects of visual attentional performance rather than a specific effect on attention per se. These data emphasize an important role for the MD in visual attention but discount a major contribution of cholinergic inputs to this region during active attentional performance.

### MD and visual attention

Pharmacological inactivation of the MD severely disrupted visual attention in the 5-CSRTT, manifested as a sharp decline in accuracy and an increased number of omitted trials, consistent with grossly impaired attention. However, response latencies were also impaired (slowed), indicating a more general impairment in attentional performance rather than deficient stimulus detection. Nevertheless, because reward collection latencies were unaffected, motor functioning and some aspects of incentive motivation appear to be less dependent on the MD. These findings were in marked contrast to the effects of AT inactivation, which disrupted accuracy only at the highest dose tested.

The putative role of the MD thalamus in attentional function is supported by research in animals (e.g. Ouhaz et al., 2015, 2018) and human imaging studies (e.g. Giraldo-Chica et al., 2018; Huang et al., 2019). The current data are consistent with previous work (Chudasama and Muir, 2001; Prasad et al., 2013), showing that either lesions of the MD or other forms of MD inactivation increase premature responses. In these studies, rodent behaviour was measured using the 5-CSRTT and rodent psychomotor vigilance test, in which increased premature responses, as a manifestation of poor impulse control, associate strongly with attentional impairment. A plausible explanation for the lack of effect on visual attention tasks in the Chudasama and Muir (2001) study is the chronic nature of excitotoxic lesions used by these investigators, which could theoretically allow compensatory mechanisms to rescue 5-CSRTT performance. Using a transient inactivation method instead, that is, brain region-specific microinfusions of muscimol and baclofen, the current study provides evidence for a critical role of the MD in visual attention processes.

### Potential role for MD-PFC interactions in visual attention

The MD sends dense projections to the PFC, specifically the cingulate cortex and lateral orbitofrontal cortex (OFC) (Parnaudeau et al., 2015). These projections contribute to cortico-thalamo-cortical circuits, with the PFC sending projections to the MD, whereas the cortico-thalamic neurons, in turn, receive direct synaptic input from thalamo-cortical neurons. However, they can also be construed as being part of cortico-striatal-thalamo-cortical loops, with a critical involvement in cognitive functions dependent on the PFC (Collins et al., 2018; Mitchell, 2015; Peters et al., 2016). Functional evidence in support of the roles of thalamocortical pathways in cognition include enhanced thalamocortical synchrony in mice performing working-memory tasks (Parnaudeau et al., 2015) and evidence that the PFC recruits the MD to enhance attentional control (Schmitt et al., 2017).

Lesions to the anterior cingulate cortex decrease accuracy while increasing omissions in the 5-CSRTT, whereas OFC lesions increase omissions without affecting accuracy (Koike et al., 2016; Mar et al., 2011). It can be speculated therefore that MD inactivation in the present study impaired visual attention by disrupting thalamo-cortical circuits linking the MD with several fronto-cortical regions. In contrast, connectivity of the AT with, for example, the hippocampus, appear to be less important for visual attentional processing in the 5-CSRTT.

### Cholinergic manipulations do not affect measures of visual attention in the 5-CSRTT

We observed no significant effect of scopolamine or mecamylamine on any measure of attention (i.e. accuracy or omissions), when either the AT or MD was inactivated. The higher doses affected other 5-CSRTT measures, including latencies and impulsive action (see below). Thus, despite using otherwise effective doses of cholinergic agents, we found no evidence for the hypothesis that cholinergic input to the thalamus modulates visual attention. This finding is at odds with evidence that cholinergic input to the MD is linked to attentional function. For example, lesions of the PPTg that deplete the cholinergic innervation of the MD profoundly impair attention performance in the 5-CSRTT (Cyr et al., 2015; Inglis et al., 2001). Despite the possibility that PPTg lesions also deplete the cholinergic innervation of the PFC, thereby impairing attention, MD lesions appear to affect attentional processing via non-cholinergic thalamocortical projections (Mar et al., 2011; Ouhaz et al., 2017).

Moreover, despite prominent neural projections from the PPTg to the thalamus, it is conceivable that cholinergic projections to other brain sites are responsible for the effects on attention following PPTg lesions. For instance, there are PPTg cholinergic fibres both in the striatum and in other dopaminergic nuclei of the midbrain (Dautan et al., 2014, 2016), with such brain regions holding established roles in mediating impulse control (Dalley et al., 2007) as well as visual attention (Boekhoudt et al., 2017). The NBM in the basal forebrain, implicated in aspects of attentional task performance in the 5-CSRTT arguably due to its cholinergic projections to the PFC, also receives cholinergic input from the PPTg (Cyr et al., 2015), again implicating cholinergic neurons originating from the PPTg in attentional task performance.

### Effects on impulsivity

Premature response in the 5-CSRTT significantly increased following reversible inactivation of the thalamus sub-regions, an effect that was partly recapitulated by mecamylamine. However, the effect was relatively modest and did not significantly differ between infusion sites (i.e. there were no dose × brain region interactions), indicating that nicotinic signaling in a diffuse area of the thalamus, including both the MD and AT, contributes to impulse control. In this context, it is worth noting that both MD afferent regions, such as the cingulate cortex and AT afferent regions, such as the dorsal hippocampus, have previously been linked to premature responses in the 5-CSRTT (Finlay et al., 2015). This could suggest that both thalamo-cortical and thalamo-hippocampal circuitry contribute to impulse control, although it should be noted that excitotoxic lesions to the MD, but not the AT, increased premature responding (Chudasama and Muir, 2001).

An area for future studies to explore is the role played by nicotinic ACh receptors in mediating impulse control within the thalamus. Whereas the present data suggest impaired function of this behavioural parameter after blocking such receptors, a previous study investigating working memory in rats observed that acute and chronic intra-MD infusions of dihydro-β-erythroidine, a α4β2 nicotinic receptor antagonist, improved working memory, an effect that is the opposite to the one seen following similar infusions into the hippocampus and amygdala (Cannady et al., 2009). This improvement was reversed by systemic nicotine, as well as by co-infusing the α7 nicotinic receptor antagonist, methyllyaconitine, indicating α4β2 and α7 may play dissociable roles in modulation of memory by the MD. Similarly, the role of nicotinic ACh receptor subtypes in impulse control also remains to be investigated.

There are some limitations of our study that merit further discussion. As only males were tested it is unclear whether our findings would generalize to female rats. However, sex differences in 5-CSRTT performance appear to be relatively minor (e.g. Anshu et al., 2017) and only apparent when task difficulty is increased (Bayless et al., 2012). In addition, no major sex-dependent morphological differences have been noted in the rat brain’s cholinergic systems (Giacobini and Pepeu, 2018), whereas, to the best of our knowledge, sexually dimorphic differences in cholinergic function have not been reported in the thalamus. Thus, it is unlikely our present findings would be markedly different in female rats; however, further investigations would be needed to verify this assertion. Rats in the present study also received multiple infusions, potentially culminating in local microglial activation. As microglia express both cholinergic and GABAergic receptors (reviewed by Liu et al., 2016), it is possible that microglia upregulation somehow interacted with the locally infused GABAergic and cholinergic agents and affected behavioural output. Additional experiments would be required to address this possibility, specifically to determine whether the essentially null findings reported for the cholinergic antagonists were not due to low statistical power for some contrasts (see Supplementary Table 1) or the presence of activated microglia in the local vicinity of the infusion sites. Finally, cell-type specificity within MD and AT could be achieved with genetically engineered techniques such as in-vivo optogenetics and chemogenetics.

## Conclusion

Here we showed that transient, reversible inactivation of the MD, but not the AT, significantly impairs sustained visual attention on a touchscreen-based version of the 5-CSRT. Our findings clearly indicate divergent contributions of these thalamic sub-regions in active attentional control and performance, presumably mediated by MD to PFC connections. Our data indicate that neither nicotinic nor muscarinic receptors modulate attentional performance at the level of the MD or AT. Nevertheless, transient inactivation of these regions increased impulsive responding, an effect that was partly recapitulated by nicotinic receptor antagonism. These findings thus reveal dissociable contributions of two major thalamic subregions in visual attentional performance and response inhibitory control.

## Supplemental Material

Suppl_Table_1 – Supplemental material for Dissociable contributions of mediodorsal and anterior thalamic nuclei in visual attentional performance: A comparison using nicotinic and muscarinic cholinergic receptor antagonists Click here for additional data file. Supplemental material, Suppl_Table_1 for Dissociable contributions of mediodorsal and anterior thalamic nuclei in visual attentional performance: A comparison using nicotinic and muscarinic cholinergic receptor antagonists by Craig P Mantanona, Tadej Božič, Yogita Chudasama, Trevor W Robbins, Jeffrey W Dalley, Johan Alsiö and Ilse S Pienaar in Journal of Psychopharmacology
