# Supplementary material for: Dissociable contributions of mediodorsal and anterior thalamic nuclei in visual attentional performance: A comparison using nicotinic and muscarinic cholinergic receptor antagonists
Source: J Psychopharmacol. 2020 Oct 24;34(12):1371–81. doi: 10.1177/0269881120965880 (PMC7708668; doi:10.1177/0269881120965880)
Supplement: Suppl_Table_1 – Supplemental material for Dissociable contributions of mediodorsal and anterior thalamic nuclei in visual attentional performance: A comparison using nicotinic and muscarinic cholinergic receptor antagonists [file Suppl_Table_1.pdf]

Supplemental Table 1. An overview of F statistics, *p* values and effect sizes (partial eta squared) from the main outcomes of the 2-way ANOVA performed for each Latin square. Where applicable, the method of transformation employed is shown.

	**Baclofen & Muscimol**		**Scopolamine**		**Mecamylamine**	
	Treatment	Treatment × Region	Treatment	Treatment × Region	Treatment	Treatment × Region
**Number of trials**	$F_{(2, 26)}$= 13.180, ***$\boldsymbol{p}$ **< 0.001**, η^2= 0.503, 1-β= 0.994; square root transformation	$F_{(2, 26)}$= 9.449, ***$\boldsymbol{p}$ **< 0.001**, η^2= 0.421, 1-β= 0.965; square root transformation	$F_{(1.365, 16.385)}$= 2.723, $\boldsymbol{p}$ **= 0.109**, η^2= 0.185, 1-β = 0.391; square root transformation	$F_{(1.365, 16.385)}$= 0.267, $\boldsymbol{p}$ **= 0.684**, η^2= 0.022, 1-β = 0.081; square root transformation	-	-
**% Accuracy**	$F_{(2, 26)}$= 23.932, ***$\boldsymbol{p}$ **< 0.001**, η^2= 0.648, 1-β= 1.000	$F_{(2, 26)}$= 5.852, **$\boldsymbol{p}$ **= 0.008**, η^2= 0.310, 1-β= 0.831	$F_{(2, 24)}$= 1.895, $\boldsymbol{p}$ **= 0.172**, η^2= 0.136, 1-β= 0.354	$F_{(2, 24)}$= 0.317, $\boldsymbol{p}$ **= 0.731**, η^2= 0.026, 1-β= 0.095	$F_{(3, 33)}$= 0.780, $\boldsymbol{p}$ **= 0.514**, η^2= 0.066, 1-β= 0.199	$F_{(3, 33)}$= 1.309, $\boldsymbol{p}$ **= 0.288**, η^2= 0.106, 1-β= 0.316
**% Omitted responses**	$F_{(2, 26)}$= 9.391, **$\boldsymbol{p}$ **= 0.003**, η^2= 0.419, 1-β= 0.901; arcsine transformation	$F_{(2, 26)}$= 4.565, *$\boldsymbol{p}$ **= 0.035**, η^2= 0.260, 1-β= 0.610; arcsine transformation	$F_{(2, 24)}$= 2.098, $\boldsymbol{p}$ **= 0.145**, η^2= 0.149, 1-β= 0.388	$F_{(2, 24)}$= 2.446, $\boldsymbol{p}$ **= 0.108**, η^2= 0.169, 1-β= 0.444	$F_{(3, 33)}$= 0.413, $\boldsymbol{p}$ **= 0.745**, η^2= 0.036, 1-β= 0.123; arcsine transformation	$F_{(3, 33)}$= 0.840, $\boldsymbol{p}$ **= 0.482**, η^2= 0.071, 1-β= 0.212; arcsine transformation
**% Premature responses**	$F_{(2, 26)}$= 4.418, *$\boldsymbol{p}$ **= 0.022**, η^2= 0.254,	$F_{(2, 26)}$= 0.616, $\boldsymbol{p}$ **= 0.548**, η^2= 0.045,	$F_{(2, 24)}$= 1.097, $\boldsymbol{p}$**= 0.350**, η^2= 0.084, 1-β= 0.220	$F_{(2, 24)}$= 1.100, $\boldsymbol{p}$**= 0.349**, η^2= 0.084,	$F_{(3, 33)}$= 3.361, *$\boldsymbol{p}$**= 0.030**, η^2= 0.234,	$F_{(3, 33)}$= 1.158, $\boldsymbol{p}$**= 0.341**, η^2= 0.095, 1-β= 0.282;

	1-β= 0.709; arcsine transformation	1-β= 0.142; arcsine transformation		1-β= 0.220	1-β= 0.709; arcsine transformation	arcsine transformation
**Perseverative responses**	$F_{(2, 26)}$= 10.913, ***$p < 0.001$, η^2= 0.456, 1-β= 0.982	$F_{(2, 26)}$= 5.273, *$p = 0.012$, η^2= 0.289, 1-β= 0.789	$F_{(2, 24)}$= 1.058, **$p = 0.363$**, η^2= 0.081, 1-β= 0.213	$F_{(2, 24)}$= 0.684, **$p = 0.514$**, η^2= 0.054, 1-β= 0.152	$F_{(3, 33)}$= 0.813, **$p = 0.496$**, η^2= 0.069, 1-β= 0.206; arcsine transformation	$F_{(3, 33)}$= 1.518, **$p = 0.228$**, η^2= 0.121, 1-β= 0.363; arcsine transformation
**Reward collection latency**	$F_{(2, 26)}$= 2.038 **p= 0.151**, η^2= 0.136, 1-β= 0.381; logarithmic transformation	$F_{(2, 26)}$= 0.723, **$p = 0.495$**, η^2= 0.053, 1-β= 0.159; logarithmic transformation	$F_{(2, 24)}$= 0.836, **p= 0.446**, η^2= 0.065, 1-β= 0.176; logarithmic transformation	$F_{(2, 24)}$= 2.276, **p= 0.124**, η^2= 0.159, 1-β= 0.417; logarithmic transformation	$F_{(3, 33)}$= 1.996, **p= 0.134**, η^2= 0.154, 1-β= 0.466; logarithmic transformation	$F_{(3, 33)}$= 0.694, **p= 0.562**, η^2= 0.059, 1-β= 0.181; logarithmic transformation
**Correct response latency**	$F_{(2, 26)}$= 3.981, *p= 0.031, η^2= 0.234, 1-β= 0.661; logarithmic transformation	$F_{(2, 26)}$= 2.886, **p= 0.074**, η^2= 0.182, 1-β= 0.515; logarithmic transformation	$F_{(2, 24)}$= 7.755, **p= 0.003, η^2= 0.393, 1-β= 0.921; logarithmic transformation	$F_{(2, 24)}$= 0.987, **p= 0.387**, η^2= 0.076, 1-β= 0.201; logarithmic transformation	$F_{(3, 33)}$= 0.502, **p= 0.683**, η^2= 0.044, 1-β= 0.141; logarithmic transformation	$F_{(3, 33)}$= 0.399, **p= 0.755**, η^2= 0.035, 1-β= 0.120; logarithmic transformation
**Incorrect response latency**	$F_{(2, 26)}$= 2.038, **p= 0.151**, η^2= 0.136, 1-β= 0.381; logarithmic transformation	$F_{(2, 26)}$= 4.321, *p= 0.024, η^2= 0.249, 1-β= 0.699; logarithmic transformation	$F_{(2, 24)}$= 1.885, **p= 0.174**, η^2= 0.136, 1-β= 0.353	$F_{(2, 24)}$= 1.855, **p= 0.178**, η^2= 0.134, 1-β= 0.348	$F_{(3, 33)}$= 0.535, **p= 0.662**, η^2= 0.046, 1-β= 0.147; logarithmic transformation	$F_{(3, 33)}$= 0.298, **p= 0.827**, η^2= 0.026, 1-β= 0.101; logarithmic transformation
